# Ranking the Leading Risk Factors for Sudden Unexpected Death in Epilepsy

**DOI:** 10.3389/fneur.2017.00473

**Published:** 2017-09-21

**Authors:** Christopher M. DeGiorgio, Daniela Markovic, Rajarshi Mazumder, Brian D. Moseley

**Affiliations:** ^1^UCLA Department of Neurology, Los Angeles, CA, United States; ^2^David Geffen School of Medicine at UCLA, Los Angeles, CA, United States; ^3^Olive View-UCLA Medical Center, Sylmar, CA, United States; ^4^Department of Neurology and Rehabilitation Medicine, University of Cincinnati, Cincinnati, OH, United Statesio

**Keywords:** epilepsy, seizures, sudden death, sudden unexpected death in epilepsy, mortality, odds ratio

## Abstract

**Background:**

Sudden unexpected death in epilepsy (SUDEP) is rare in well-controlled epilepsy. However, SUDEP is a common cause of death in drug-resistant epilepsy. Over the last 30 years, multiple cohort and population studies have identified clinical risk factors associated with an increased risk for SUDEP.

**Objective:**

To identify and rank the leading SUDEP risk factors from major cohort and population-based studies. The incidence of SUDEP is also evaluated in special clinical situations, including antiepileptic drug treatment, epilepsy surgery, devices, and assignment to placebo in clinical trials.

**Methods:**

A PubMed search for English language human cohort studies for the terms Sudden, Death, and Epilepsy was performed for the years 1987–2017. Risk factors for SUDEP were identified and ranked by the weighted log adjusted odds ratio (OR)/relative risk ratio (RR).

**Findings:**

The top 10 leading risk factors ranked from highest to lowest log adjusted OR/RR are the following: ≥3 GTC seizures per year; ≥13 seizures in the last year; No Antiepileptic Drug (AED) treatment; ≥3 AEDs; ≥3 GTCs in the past year; 11–20 GTC seizures in the last 3 months; age of onset 0–15 years old; IQ < 70; 3–5 AED changes in the last year; ≥3 AEDs. Two risk factors from separate sources (≥3 GTC seizures and ≥3 AEDs) occur twice in the top 10 risk factors.

**Conclusion:**

The top 10 risk factors for SUDEP are identified and ranked. A ranking of the top risk factors could help clinicians identify patients at highest risk for SUDEP.

## Introduction

Sudden unexpected death in epilepsy (SUDEP) is an important cause of death in people with epilepsy (1, 2). Average incidence is 0.2 per 1,000 persons/year in children and 1.0 per 1,000 persons/year in adults (1, 3, 4). The recent American Academy of Neurology guideline reassures patients and families that SUDEP is rare in people with well-controlled epilepsy (1). However, SUDEP is relatively common in persons with drug-resistant epilepsy, accounting for 14.7–17.4% of deaths with an incidence of 2.46–5.94 per 1,000 persons/year (5–8). Risk factors associated with increased risk for SUDEP risk are frequent GTC seizures, polytherapy, early onset, long duration of epilepsy, frequent antiepileptic drug (AED) changes, and low IQ (9–13).

The purpose of this report is to review and rank the leading SUDEP risk factors from studies published in core clinical and epilepsy journals from 1987 to 2017. We also review the incidence of SUDEP in specific clinical situations. The authors hope this report will provide an accessible reference for clinicians to help identify persons at highest risk for SUDEP.

## Methods

A PubMed search for all English Language Human publications from 1987 to 2017 was performed for the terms “Sudden,” “Death,” and “Epilepsy” in journal titles. This search yielded 310 publications. An identical similar search including the term “Population” or “Cohort” yielded 64 and 30 publications, respectively. A search of core clinical journals for these terms yielded 20 and 18 publications, respectively. Both prospective and retrospective studies were included and duplicate studies were eliminated. Publications from core clinical journals and core epilepsy journals indexed in PubMed were included.

Risk factors from cohort studies using matched control subjects and analyzed using multivariate or univariate analysis were included. The crude and adjusted odds ratios or relative risk ratios (ORs or RRs) from these studies were analyzed. For ranking of risk factors for inclusion in Table 1, the adjusted log OR/RR was used. The weighted log of the adjusted OR was calculated by multiplying the adjusted log OR × 1/SE to adjust for the size and variability of the point estimates of the source studies. The top 10 SUDEP risk factors were then ranked by the weighted log OR or RR. Variables accounting for the adjusted OR are listed in Table 1 for each individual study, and include: age, gender, seizure frequency, geographic region, epilepsy duration, and data source.

**Table 1:** Top 10 risk factors for SUDEP sorted by the weighted log OR estimate.

Ranking by weighted log OR	Risk factor	Adjusted OR	Lower CI	Upper CI	Weighted log OR [*adjusted log OR × 1/SE*]	Reference	Study methodology/OR adjustments
1	Three or more GTC seizures per year (versus 0 seizures)	15.46	9.92	24.10	12.10	Hesdorffer et al. (13)	Pooled analysis from four cohort studies. Risk factors were examined using logistic regression. ORs were adjusted for data source, gender, age at death, and epilepsy duration
2	≥ 13 seizures of any type in the last year (versus 0–2 seizures)	9.15	3.26	25.68	4.20	Nilsson et al. (9)	Cohort Study: relative risk ratios (RRs) were calculated using multiple logistic regression. Each index case of SUDEP was compared with three living controls matched for year of birth, sex, and assessment period randomly selected from the study. ORs were adjusted for population age, gender, and seizure frequency
3	No AED treatment (versus one or two AEDs)	21.70	4.40	106.00	3.78	Langan et al. (11)	Cohort Study: ORs were calculated using index SUDEP case compared with 4 randomly selected controls from patients with epilepsy enrolled in the MRC General Practice Research Framework. ORs were adjusted for age, geographic region, and seizure frequency
4	Three AEDs (versus 1)	8.09	2.28	28.62	3.24	Nilsson et al. (9)	Cohort Study: relative RRs were calculated using multiple logistic regression. Each index case of SUDEP was compared with three living controls matched for year of birth, sex, and assessment period randomly selected from the study. ORs were adjusted for population age, gender, and seizure frequency
5	Three or more GTC Seizures in past year (versus 0)	7.00	2.00	24.20	3.04	Walczak et al. (10)	Cohort Study: ORs were calculated using logistic regression. Index case of SUDEP compared with 4 randomly selected controls also enrolled in prospective study at 3 Midwest US epilepsy centers. ORs were adjusted for seizure frequency
6	11–20 GTC Seizures in the last 3 months (versus 0–5)	19.40	1.70	226.00	2.39	Langan et al. (11)	Cohort Study: ORs were calculated using index SUDEP case compared with 4 randomly selected controls from patients with epilepsy enrolled in the MRC General Practice Research Framework. ORs were adjusted for age, geographic region
7	Age at onset 0–15 (versus >45)	5.04	1.26	20.19	2.29	Nilsson et al. (9)	Cohort Study: relative RRs were calculated using multiple logistic regression. Each index case of SUDEP was compared with three living controls matched for year of birth, sex, and assessment period randomly selected from the study. ORs were adjusted for population age, gender, and seizure frequency
8	IQ < 70	4.60	1.20	18.00	2.23	Walczak et al. (10)	Cohort Study: ORs were calculated using logistic regression. Index case of SUDEP compared with 4 randomly selected controls also enrolled in prospective study at 3 Midwest US epilepsy centers. ORs were adjusted for seizure frequency
9	3–5 changes in AEDs per year (versus 0)	4.02	1.14	14.21	2.16	Nilsson et al. (9)	Cohort Study: relative RRs were calculated using multiple logistic regression. Each index case of SUDEP was compared with three living controls matched for year of birth, sex, and assessment period randomly selected from the study. ORs were adjusted for population age, gender, and seizure frequency
10	≥3 AEDs last visit (versus 0–2)	3.00	1.00	9.20	1.96	Walczak et al. (10)	Cohort Study: ORs were calculated using logistic regression. Index case of SUDEP compared with 4 randomly selected controls also enrolled in prospective study at 3 Midwest US epilepsy centers. ORs were adjusted for seizure frequency

*The weighted log OR is defined as the log adjusted OR × 1/SE. CI are reported*.

*OR, odds ratio; CI, confidence intervals; SUDEP, sudden unexpected death in epilepsy; AED, antiepileptic drug*.

## Results

**Figure 1:**
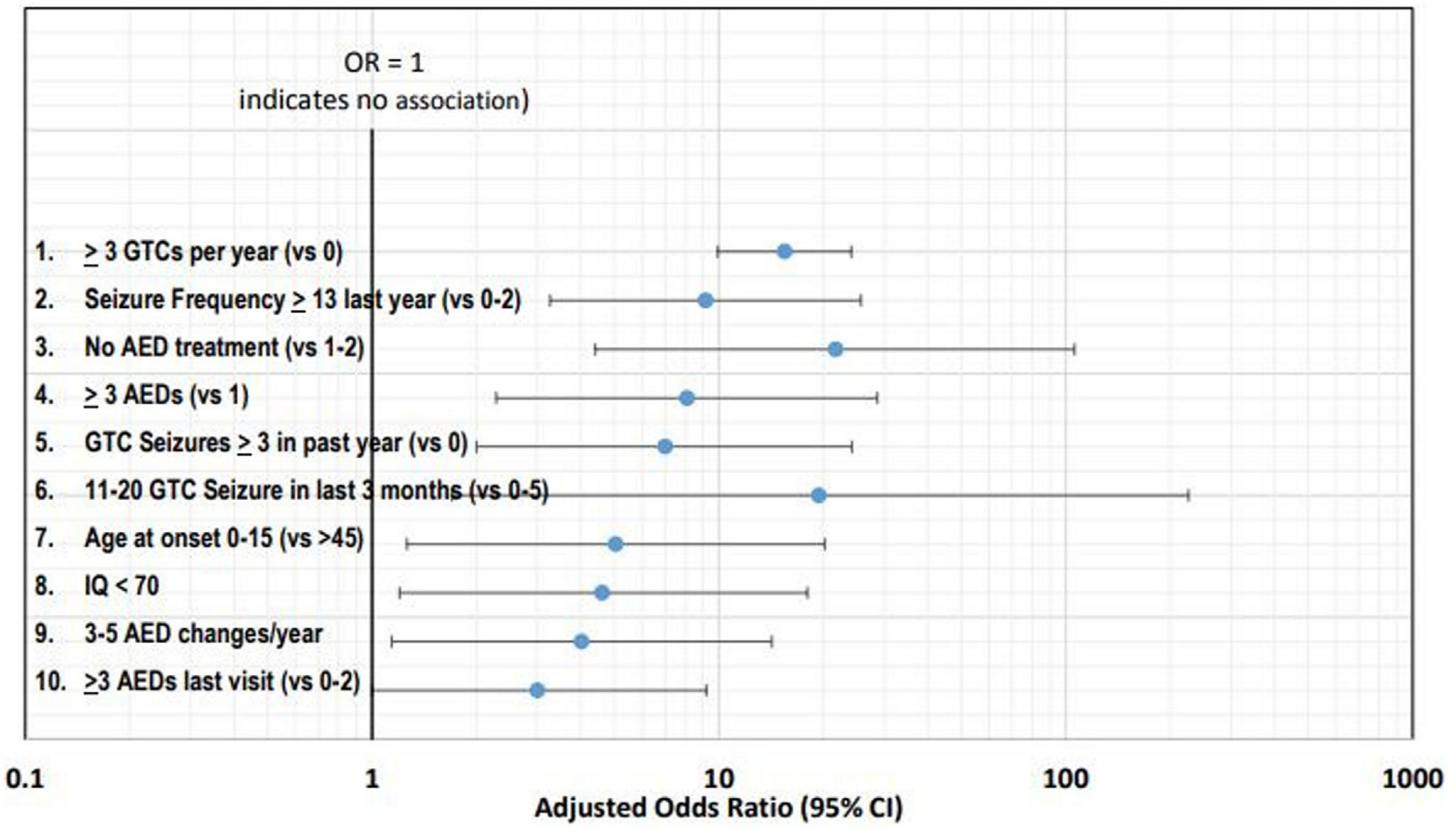
Forest plot of the 10 leading risk factors by adjusted odds ratios (ORs) with corresponding 95% confidence intervals (CI).

Table 1 summarizes the 10 leading clinical risk factors ranked by the weighted log of the adjusted OR or RR. Figure 1 graphically displays the adjusted OR/RR for these 10 risk factors with their corresponding 95% confidence intervals (CI). The top 10 leading risk factors by weighted log OR/RR are the following: #1 ≥3 GTC seizures per year (versus 0, Hesdorffer et al.) (13); #2 ≥13 seizures (of any type) in the last year (versus 0–2); #3 No AED treatment (versus 1–2 AEDs); #4 ≥3 AEDs (versus 1); #5 ≥3 GTCs in the past year (versus 0, Walczak et al.) (10); #6 11–20 GTC seizures in the last 3-months (versus 0–5); #7 age of onset 0–15 years old (versus > age 45); #8 IQ < 70; #9 3–5 AED changes/year in the last year; #10 ≥3 AEDs (versus 0–2) (8–11, 13).

### Specific Clinical Scenarios

#### GTC Seizures and Overall Seizure Frequency

GTC and frequent GTC seizures are consistently identified as the leading risk factors for SUDEP with the highest OR (10–13). GTC seizures account for 3 of the 10 leading risk factors reported in Table 1. Three or more GTC seizures in the last year (reported by Hesdorffer et al.) ranks as the leading risk factor overall and are repeated again as #5 (also reported by Walczak et al.) (10, 13). High seizure frequency of any type is also an important risk factor for SUDEP (9, 10). In their study of 6,880 subjects, Nilsson et al. reported that 13–50 seizures/year and >50 seizures/year were associated with SUDEP (crude OR of 8.64 and 10.16, respectively) (9). Similarly, Walczak et al. reported that >50 seizures of any type per month were associated with risk of SUDEP with an OR of 11.5 (CI 1.3–99.3) (10).

#### AED Therapy

Antiepileptic drug therapy and polytherapy have been extensively studied in relation to SUDEP risk (14–16). Hesdorffer et al. evaluated four large cohort studies of SUDEP, and found the risk of SUDEP in patients treated with monotherapy versus no treatment was reduced for phenytoin, carbamazepine, and valproic acid (OR ranged from 0.5 to 0.7, CI 0.2–1.6) (16).

Since CI were wide and statistical significance was not reached, these results do not provide compelling evidence of a protective effect of AED therapy against SUDEP (14–16). However, no AED treatment in patients with epilepsy has been identified as a risk factor for SUDEP and is listed in Table 1 (11).

Polytherapy accounts for 2 of the top 10 leading risk factors for SUDEP. Nilsson et al. reported that three or more AEDs were associated with an increased risk of SUDEP (9). In their series, 12 of 57 (21%) subjects who died of SUDEP were taking at least 3 AEDs, versus 7 of 171 controls (4%). Walczak found a moderate association between SUDEP and two or more AEDs (10). However, Hesdorffer et al. in their 2012 meta-analysis of published case–control studies, when adjusting for the number of GTC seizures, found a reduced association between SUDEP risk and polytherapy (16). It is likely that the risk of polytherapy is more a reflection of severe drug resistance and high seizure frequency, rather than the risk of AEDs *per se* (16).

Frequent changes in AED dose are also a risk factor. Nilsson et al. found that frequent AED dose changes were associated with the risk of SUDEP (9). The RR associated with three or more changes in AED dose in the last year was 6.08 (CI 1.99–18.56) (9).

Randomization to placebo clinical AED trials is associated with a very high risk of SUDEP. Ryvlin et al. reported an analysis of 20,101 patients enrolled in 112 randomized controlled AED trials (17). Fourteen of 7,678 patients randomized to placebo died of SUDEP, versus only 3 of 3,297 patients randomized to effective doses of investigational AEDs (17). The incidence of SUDEP was 6.9 per 1,000 patient years for those randomized to placebo, versus only 0.9 per 1,000 patient years assigned to effective doses of AEDs. Overall, the relative risk of SUDEP was 7.5 times higher in those assigned to placebo than those randomized to active treatment arms (17).

#### Resective Epilepsy Surgery

Resective epilepsy surgery is associated with reductions in mortality, especially in those who become seizure free. In 1999, Sperling et al. reported a cohort of 393 drug-resistant patients who underwent various epilepsy surgical procedures over the period 1986 to 1996 (18). The mortality of those who were not seizure free was 5.7% (CI 2.9–9.9%) for a mortality rate of 13.7 per 1,000 persons/year (18). This is substantially higher than the mortality rate of 0 per 1,000 person/year for those patients who were seizure free during the follow-up period (CI 0.0–1.8%) (18). Six of 11 subjects who died during follow-up died of probable or definite SUDEP (54.5%) (18). Of the 6 subjects who died of SUDEP, 5/6 (83%) were not seizure free, indicating that seizure freedom after epilepsy surgery is a factor in the risk for SUDEP (18). Later in 2016, Sperling et al. reported the mortality in a larger cohort of 1,110 drug-resistant patients evaluated for epilepsy surgery (19). The cohort consisted of 1,006 patients who underwent epilepsy surgery, and 104 patients who underwent pre-surgical evaluation but were treated with medical therapy only. In the 104 patients treated with medical therapy only, the mortality rate was 25.3 per 1,000 persons/year versus 8.6 per 1,000 persons/year for surgically treated patients (19). Overall, the SUDEP rate for surgically treated patients was low: 15 died of SUDEP in 8,126 persons/year (incidence = 0.6 per 1,000 persons/year) (19).

#### Devices: Vagus Nerve Stimulation (VNS) and Responsive Neurostimulation (RNS)

Annegers et al. first reported SUDEP rates with VNS therapy (20). The incidence of SUDEP in their initial cohort of 1,891 patients with drug-resistant epilepsy was 4.1 per 1,000 persons/year (20). Later, in a follow-up study, Annegers reported that the incidence of SUDEP dropped from 5.5 per 1,000 persons/year to 1.7 per 1,000 persons/year after two years of VNS therapy (21). More recently, Granbichler et al., in a series from Kings College, reported SUDEP rates in persons with drug-resistant epilepsy treated with VNS over a 15-year period (1995 through 2010). They reported SUDEP rate of 3.7 per 1,000 persons/year (22). It is currently unknown how long-term VNS therapy exerts its potential protective effect on SUDEP (22). In RNS, Heck et al. and Bergey et al. reported four probable or definite SUDEP cases, representing an incidence of 3.5 per 1,000 persons/year (23, 24). This rate is lower than the 25.3 per 1,000 persons/year reported by Sperling et al. in epilepsy surgical candidates treated with medical therapy only (19, 23, 24).

## Discussion

In this review, we identify the leading risk factors for SUDEP from published cohort studies of SUDEP and rank them by the weighted log OR. The 10 leading risk factors for SUDEP ranked by weighted log OR are the following: #1 ≥3 GTC seizures per year (versus 0); #2 ≥13 seizures of any type in the last year (versus 0–2); #3 No AED treatment (versus 1–2 AEDs); #4 ≥3 AEDs (versus 1); #5 ≥3 GTCs in the past year (versus 0); #6 11–20 GTC seizures in the last 3 months (versus 0–5); #7 age of onset 0–15 years old (versus > age 45); #8 IQ < 70 (mental retardation or developmental delay); #9 3–5 AED changes/year in the last year; #10 ≥3 AEDs (versus 0–2) (8–11, 13).

We also review the incidence and risk of SUDEP in specific clinical scenarios. For the drug-resistant patient, resective epilepsy surgery imparts a protective effect versus medical therapy alone (18, 19). This is especially true when patients are seizure free after surgery (18, 19). VNS may also be associated with long-term reductions in SUDEP risk. The data on RNS are still early. However, VNS and RNS may be associated with lower mortality rates than surgical candidates treated with medical therapy only (18–24).

Polytherapy (defined as ≥3 AEDs), frequent AED changes (3–5 per year), and assignment to placebo during clinical trials are associated with increases in SUDEP risk (9, 17). However, the role of polytherapy in SUDEP risk is controversial (16). Hesdorffer found that when the risk of polytherapy, when adjusted for the frequency of GTC seizures is diminished (16). The OR for polytherapy (>3 AEDs) when adjusted for the frequency of GTC seizures is reduced from 2.8 to 1.4 (16). Supporting a diminished role for polytherapy in SUDEP risk is the Ryvlin et al.’s study of the risk of investigational AEDs versus placebo (17). Patients on effective investigational AEDs were on multiple AEDs, yet their risk was lower than those on placebo (17).

A better understanding of the leading risk factors for SUDEP could help clinicians identify patients at highest risk. For these patients, protective measures such as listening devices, nocturnal supervision, or seizure detection devices could be considered (25, 26). Langan et al. in their large UK study reported that listening devices and nocturnal supervision reduce the risk for SUDEP up to 80–90% (11, 25). Strategies have been published that could help physicians intervene to reduce the risk of SUDEP. However, their efficacy is unproven (25, 26). http://Dannydid.org is a non-profit organization committed to reducing the risk of SUDEP, and is a good resource for patients, families, and clinicians.

Clinicians should work diligently to aggressively control GTC seizures and make patients seizure free whenever possible. Since epilepsy surgery imparts a significant reduction in mortality compared with medical therapy in potential surgical candidates, efforts to explore surgical interventions should be pursued when feasible.

Community education can be expanded to inform people with epilepsy about the risks associated with SUDEP (25). Pharmaceutical companies and regulatory bodies could change the structure of clinical trials to employ novel designs that reduce exposure to placebo. Such designs could include the use of historic controls or include robust escape criteria to allow exit for placebo patients when seizures worsen.

Risk inventories should be updated to include risk factors with the highest OR. The top 10 risk factors could be included in new SUDEP risk factor inventories, such as the SUDEP-7 (27). The original SUDEP-7 was sourced from a single prospective study reported by Walczak et al. and does not include many of the risk factors identified in Table 1 (10, 27, 28)^1^. A revision to the SUDEP risk inventory is under consideration (27, 28).

This analysis has limitations. Risk factors were derived from different studies of variable sizes, ages, and cohorts. There were variations in statistical analysis and matching techniques (four matched controls or three matched controls). The incidence of SUDEP and numbers of cases of SUDEP varied significantly from study to study. For example, there were 20 cases of SUDEP in the Walczak et al.’s study, yet 154 cases in the Langan et al.’s study (10, 11). CI were wide for many ORs and RRs, which likely reflected the relatively rare nature of SUDEP.

In conclusion, the recent AAN guideline appropriately reassures persons with well-controlled epilepsy and their families that SUDEP is rare (1). However, those with one or more risk factors listed in Table 1 should be considered at higher risk for SUDEP. Recognition of those at high risk could lead to education and individualized interventions to reduce the risk of SUDEP.

## Author Contributions

CD served as the lead author. RM helped in the concept of ranking and odds ratios, BM made several additions and contributions to the manuscript, and DM analyzed the statistics from the references cited in Table 1.

## Conflict of Interest Statement

The authors declare that the research was conducted in the absence of any commercial or financial relationships that could be construed as a potential conflict of interest.

## Footnotes

^1^Available from: http://Dannydid.org
